# MALAT-1 Expression Correlates with Prognosis in Non-Small-Cell Lung Carcinoma: A Systematic Review and Meta-analysis

**DOI:** 10.1155/2021/5424623

**Published:** 2021-11-23

**Authors:** Ran Ran, Jian-wei Jin, Wei-ping Zhang

**Affiliations:** Department of Oncology, The Third Affiliated Hospital of Zhejiang Chinese Medical University, Hangzhou, Zhejiang, China

## Abstract

**Background:**

Non-small-cell lung carcinoma (abbreviated as NSCLC) progresses rapidly and lacks appropriate biological markers. Recent studies have shown that long noncoding RNA metastasis-associated lung adenocarcinoma transcript 1 (MALAT-1) has potential application value for clinically diagnosing lung carcinoma. Thus, this study conducted a systematic review and meta-analysis for assessing if MALAT-1 has a relationship to NSCLC outcome.

**Methods:**

This study conducted the search of China National Knowledge Infrastructure, China Science and Technology Journal, SinoMed, EMBASE, Cochrane library, Web of Science, Wanfang database, and PubMed from inception to September, 1, 2021. The published article about MALAT-l expression for NSCLC patients was analyzed. We used combined hazard rates under the confidence interval of 95% for examining the relationship of MALAT-l and NSCLC.

**Results:**

In this meta-analysis, we found that 10 studies were included, and MALAT-1 expressions were distinctly related to an unfavorable overall survival (HR: 2.34 (1.65, 3.33); I2 = 76%). Considering the merger's clinical heterogeneity, for meta-analysis, we used the random-effects method.

**Conclusion:**

Overexpression of MALAT-1 showed correlations to the less effective outcome of NSCLC. MALAT-1 might be a new NSCLC prognosis marker.

## 1. Introduction

Lung carcinoma is a highly common carcinoma with high mortality, in accordance with the latest data released by the World Health Organization's International Agency for Research on Carcinoma in 2020 [1]. It falls to non-small-cell lung carcinoma (NSCLC) and small cell lung carcinoma [2]. NSCLC takes about 80 percentage of all lung carcinomas [3]. Due to the lack of highly sensitive and specific biological indicators for early diagnosis, the 5-year survival rate after diagnosis is no more than 20% [4, 5]. Early NSCLC can get prognosis by surgical resection; however, most patients are in a serious stage when diagnosed, and the 5-year survival in total is only about 15% [6, 7]. Early NSCLC patients' clinical symptom is nonspecific. More than 65% of patients suffered from advanced diseases during diagnosis and missed the best surgery chance [8, 9]. Although there are some clinical markers for the diagnosis of NSCLC, such as carcinoembryonic antigen and cytokeratin 19 fragments. However, their sensitivity and specificity are low.

Long non-coding RNA (lncRNA) covers over 200 nucleotides [10]. As reported extensively, lncRNA is critical to the following: cell differentiation regulation, epigenetic regulation, and cell cycle regulation, turning into a genetic study hotspot [11, 12]. MALAT-1 is the first discovered lncRNA in NSCLC [13]. In recent years, several studies have reported the tumor-related functions of MALAT-1 in several types of tumors. For instance, MALAT-1 expression was distinctly upregulated in colorectal carcinoma, and its knockdown suppressed colorectal carcinoma cells' metastasis and proliferation via sponging miR-203/DCP1A axis [14]. MALAT-1 was observed to exhibit a higher level in pancreatic carcinoma and promote cellular growth and metastasis of pancreatic carcinoma cells [15]. It was also observed that MALAT-1 knockdown suppressed autophagy via decreasing HMGB1 in multiple myeloma [16]. In NSCLC, MALAT-1 was highly expressed and promoted EMT and development of NSCLC cells [17]. These findings highlighted the critical effects of MALAT-1 in the progression of different types of tumors. Moreover, more and more evidence has shown that MALAT-1 has potential application value in the clinical diagnosis of lung carcinoma, which suggests that MALAT-1 can be used as one of the indicators for the early NSCLC diagnosis [18, 19].

However, due to the limitations such as small sample size of single research, there are great differences between different research results. The clinical application value of MALAT-1 has not been widely concerned. This study intends to systematically evaluate the prognostic significance of lncRNA MALAT-1 expression for NSCLC so as to provide evidence-based medical reference for further study of MALAT-1 and development of new tumor markers at the early stage.

## 2. Methods

### 2.1. Searching Strategies

Literature searching was carried out about relationship between lncRNAs and the diagnostic and prognostic of NSCLC using the following databases up to 1st September, 2021: Wanfang database, Web of Science, Cochrane library, EMBASE, PubMed, SinoMed, China Science and Technology Journal, and China National Knowledge Infrastructure. The specific retrieval strategies of PubMed are as follows:

non small cell lung carcinoma/exp
nsclc…ti,ab
lung carcinoma∗:ti,ab
lung carcinom∗:ti,ab
lung neoplasm∗:ti,ab
lung tumor∗:ti,ab
lung tumour∗:ti,ab
non small cell∗:ti,ab
nonsmall cell∗:ti,ab
or (1)–(9)
long non coding RNA/exp
lncRNA/exp
or (11)–(12)
(10) and (13)

### 2.2. Inclusion Standards

The inclusion standards are as follows: (1) all the patients were diagnosed as NSCLC; (2) the research content is about the application or evaluation of circulating (serum, plasma, or whole blood) MALAT-1 in the diagnosis of lung carcinoma; (3) the study needs to provide sufficient diagnostic indexes to meet the data extraction and statistical investigation; (4) using quantitative PCR to present MALAT-1 expression with NSCLC patients; and (5) researches assessed the correlations of overall survival (OS) and the expression of MALAT-1 in terms of NSCLC patients.

### 2.3. Exclusion Standards

The standards in terms of exclusion included (1) duplicate articles; (2) animal experiment, conference summary, case report, and review; (3) no data can be extracted; and (4) no OS data could be analyzed.

### 2.4. Data Extraction

Given exclusion and inclusion standards, two reviewers (Ran Ran and Jian-wei Jing) evaluated all the entered documents and analyzed the data independently. For any disagreement, two researchers carried out the discussion on the result. In addition, the third reviewer (Wei-ping Zhang) resolved differences in case of data contradiction. Data extraction contents are as follows: (1) the basic characteristics of the included study, including the first author, publication year, publication country, language, and number of cases; (2) the required data were directly extracted from the text or obtained from the survival curve by using the Engauge digitizer 4.1 software, and the risk ratio of overall survival (OS) was obtained by calculation for prognostic investigation.

### 2.5. Literature Quality Evaluation

Newcastle-Ottawa Scale was used to achieve quality assessment. The articles from 0 to 9 were classified in accordance with Newcastle-Ottawa Scale judgment standards.

### 2.6. Statistical Investigation

We calculate HR data according to the survival investigation curve using the following method: (1) we recorded the HR and 95% CI from literature directly. (2) According to the survival curve, this study employed the Engauge digitizer 4.1 software for reading survival data, and then, this study determined the HR and 95% credibility interval. With the use of the Review Manager Software (Rev Man version 5.3, Cochrane Collaboration, Oxford, UK), we carried out the statistical investigations. Q statistics were utilized to estimate heterogeneity, and I2 was employed for quantifying the impact of heterogeneity. I2 data of 25, 50, and 75% were, respectively, used as evidence of low, moderate, and high heterogeneity. When I2 values reach over 50%, this study will adopt a random effect method for merging HR value. Given heterogeneity test's results, fixed/random effect model was employed to achieve meta-analysis. In addition, this study conducted a Subdivided group investigation for finding potential sources of heterogeneity. To achieve meta-analysis, the RevMan 5.3 software was employed.

## 3. Result

### 3.1. Literature Search

**Figure 1:**
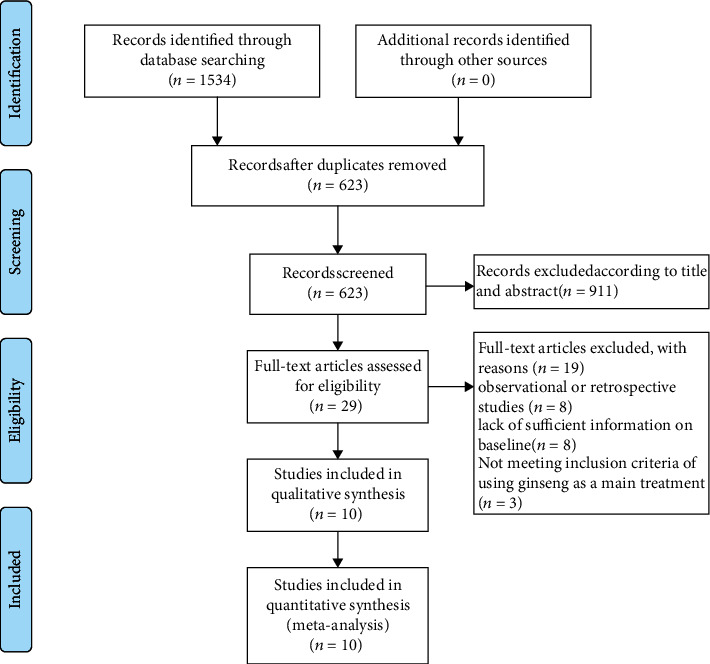
Flow diagram of the identification and selection of studies.

A total of 1534 were potentially eligible articles searched from the databases. Through reading the full text and abstract, 10 were finally selected according to the inclusion and exclusion standards of this study. A flow chart of the screening process for the articles is shown in Figure 1.

### 3.2. Characteristics of Involved Articles

10 articles including 1250 patients were included in this study. These involved articles were from China and Germany. The Newcastle-Ottawa Scale of quality evaluation was completed by two researchers independently (Ran Ran and Wei-ping Zhang). The average score of the literature was 6.4.  Table 1 elucidates the mentioned characteristics.

### 3.3. Association between lncRNA MALAT-l Expression

**Figure 2:**
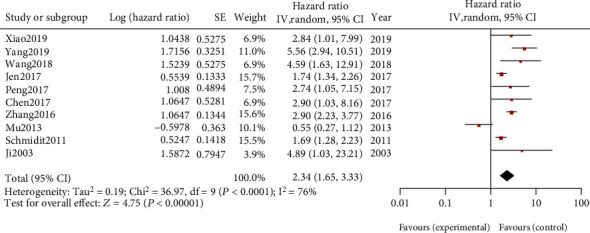
Forest plot for the relationship between MALAT-l expression and overall survival.

10 articles include 1250 participants in the meta-analysis reporting OS (Figure 2). Considering of the huge heterogeneity (I2 = 76%), a random-effect model was applied. The combined HR and 95% CI due to heterogeneity were included (HR = 2.34, 95% CI: 1.65-3.33, *P* < 0.0001), which is statistically significant. We found that the result of Mu2013 completely opposed to other studies. We consider that this study may be a source of heterogeneity. The result suggests that MALAT-1 may be related to the prognosis of NSCLC (Figure 2).

### 3.4. Publication Bias

**Figure 3:**
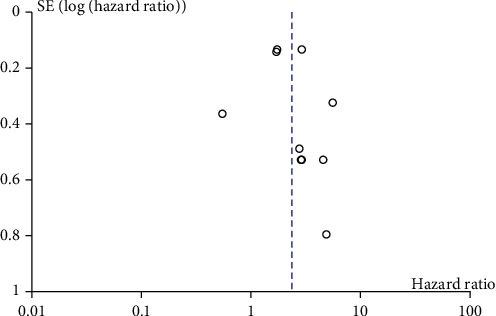
Funnel plot for the assessment of potential publication bias of the diagnostic studies.

The funnel plot showed asymmetry, and the involved articles were mostly small samples. Therefore, there was publication bias (Figure 3).

### 3.5. Subdivided Group Investigation of lncRNA MALAT-l Expression

**Figure 4:**
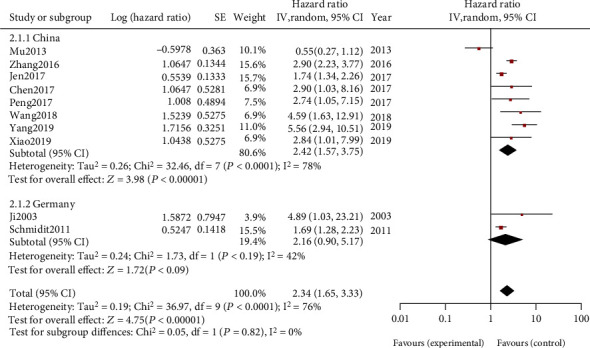
Forest plot for the association between MALAT-l expression levels and overall survival of patients from different countries.

In order to explore the source of heterogeneity, we conducted Subdivided group investigation according to the different region (China and Germany). The results showed that China OS (HR = 2.42, 95% CI: 1.57-3.75, *P* < 0.0001, I2 = 78%) and Germany OS (HR = 2.16, 95% CI: 0.90-5.17, *P* < 0.0001, I2 = 42%), which are both statistically significant (Figure 4). It suggested that MALAT-l expression was related with NSCLC in both China and Germany.

## 4. Discussion

NSCLC is a malignant tumor with the maximal incidence and mortality in the world [20, 21]. LncRNA is increasingly critical to tumorigenesis and development [22, 23]. It can regulate gene expression at multiple levels and participate in tumor growth, metastasis, and invasion [24, 25]. In order to assess the correlation between MALAT-1 and survival time of cases, an in situ hybridization was performed in 352 cases of non-small-cell lung carcinoma. It was found that the high expression of MALAT-1 in squamous cell lung carcinoma was closely related to the poor prognosis of cases [26]. In order to explore the mechanism of MALAT-1 promoting lung carcinoma cell migration, a study used siRNA to interfere with MALAT-1 expression and found that cell motility decreased significantly. By analyzing the differential gene expression of cells before and after interference, it was found that MALAT-1 controlled the migration capability of lung carcinoma cells by regulating the expression of motion-related genes [27]. It was also found that the expression of MALAT-1 in NSCLC noticeably exceeded that in adjacent normal tissues. When siRNA interference was achieved, the migration ability of lung carcinoma cells and the tumorigenicity of nude mice decreased noticeably. It showed that MALAT-1 has the ability to promote the formation of lung carcinoma. Therefore, we performed meta-analysis to assess the prognosis significance of MALAT-1 expression in NSCLC.

As impacted by the clinical heterogeneity of the merger, the random-effect model investigation was used for meta-analysis. 10 studies were covered in the present meta-analysis, and MALAT-1 expression showed correlations with OS (HR: 2.34 (1.65, 3.33); I2 = 76%). The Subdivided group investigation result shows that China OS (HR = 2.42, 95% CI: 1.57-3.75, *P* < 0.0001, I2 = 78%) and Germany OS (HR = 2.16, 95% CI: 0.90-5.17, *P* < 0.0001, I2 = 42%), which are both statistically significant. It suggests that MALAT-l expression is related to NSCLC in both China and Germany. In summary, our study suggests that MALAT-1 may play a significant role in NSCLC.

This study provides a certain value for future research on the prognostic significance of long noncoding RNA MALAT-1 expression for NSCLC. However, our study has several limitations. First, the experimental sample size is small and cannot provide high-quality evidence. Secondly, Subdivided group investigation and sensitivity investigation were not conducted to evaluate high heterogeneity due to the small sample. Lastly, the involved articles were conducted in China or Germany. Ethnic differences may lead to different investigation indicators.

## 5. Conclusion

The increase of MALAT-1 in NSCLC patients showed correlations to the prognosis of NSCLC. MALAT-1 is likely to be a promising NSCLC prognosis biological marker.

## Figures and Tables

**Table 1:** Characteristics of the studies included in the meta-analysis.

Study	Country	Language	Sample size	HR	95% CI	NOS scores
Yang, 2019 [28]	China	English	326	5.56	2.94, 10.00	6
Xiao, 2019 [29]	China	English	39	2.84	1.01, 9.17	6
Wang, 2018 [30]	China	English	56	4.56	1.69, 12.46	6
Peng, 2017 [31]	China	English	60	1.74	1.34, 2.65	7
Chen, 2017 [32]	China	English	42	2.8	1.03, 8.14	7
Jen, 2017 [33]	China	English	124	2.74	1.05, 5.35	6
Zhang, 2016 [34]	China	English	125	1.77	1.36, 2.75	7
Mu, 2013 [35]	China	English	76	0.55	0.27, 0.99	6
Schmidt, 2011 [26]	China	English	352	1.69	1.28, 2.24	7
Ji, 2003 [36]	China	English	50	4.89	1.03, 14.40	6

## Data Availability

All data generated or analyzed during this study are included in this article.
